# Molecular Basis for Converting (2*S*)-Methylsuccinyl-CoA Dehydrogenase into an Oxidase

**DOI:** 10.3390/molecules23010068

**Published:** 2017-12-28

**Authors:** Simon Burgener, Thomas Schwander, Elvira Romero, Marco W. Fraaije, Tobias J. Erb

**Affiliations:** 1 Department of Biochemistry and Synthetic Metabolism, Max Planck Institute for Terrestrial Microbiology, Karl-von-Frisch-Str. 10, 35043 Marburg, Germany; simon.burgener@mpi-marburg.mpg.de (S.B.); thomas.schwander@mpi-marburg.mpg.de (T.S.); 2 Molecular Enzymology Group, Groningen Biomolecular Sciences and Biotechnology Institute, University of Groningen, Nijenborgh 4, 9747 AG Groningen, The Netherlands; e.romero.guzman@rug.nl (E.R.); m.w.fraaije@rug.nl (M.W.F.); 3 LOEWE Center for Synthetic Microbiology, University of Marburg, 35043 Marburg, Germany

**Keywords:** acyl-CoA dehydrogenase, acyl-CoA oxidase, enzyme engineering, flavin adenine dinucleotide

## Abstract

Although flavoenzymes have been studied in detail, the molecular basis of their dioxygen reactivity is only partially understood. The members of the flavin adenosine dinucleotide (FAD)-dependent acyl-CoA dehydrogenase and acyl-CoA oxidase families catalyze similar reactions and share common structural features. However, both enzyme families feature opposing reaction specificities in respect to dioxygen. Dehydrogenases react with electron transfer flavoproteins as terminal electron acceptors and do not show a considerable reactivity with dioxygen, whereas dioxygen serves as a bona fide substrate for oxidases. We recently engineered (2*S*)-methylsuccinyl-CoA dehydrogenase towards oxidase activity by rational mutagenesis. Here we characterized the (2*S*)-methylsuccinyl-CoA dehydrogenase wild-type, as well as the engineered (2*S*)-methylsuccinyl-CoA oxidase, in detail. Using stopped-flow UV-spectroscopy and liquid chromatography-mass spectrometry (LC-MS) based assays, we explain the molecular base for dioxygen reactivity in the engineered oxidase and show that the increased oxidase function of the engineered enzyme comes at a decreased dehydrogenase activity. Our findings add to the common notion that an increased activity for a specific substrate is achieved at the expense of reaction promiscuity and provide guidelines for rational engineering efforts of acyl-CoA dehydrogenases and oxidases.

## 1. Introduction

Acyl-CoA dehydrogenases (ACADs) are flavoproteins that catalyze the flavin adenosine dinucleotide (FAD)-dependent oxidation of α,β-carbon bonds in acyl-CoA thioesters. ACADs are found in all kingdoms of life and are part of various metabolic pathways, such as amino acid oxidation, choline metabolism and most prominently, the initial step in fatty acid β-oxidation [1]. ACADs transfer the electrons from the substrate to an electron transfer flavoprotein (ETF), which in turn funnels the electrons into a membrane bound electron transport chain and from there to the final electron acceptor [2,3].

Overall, the reaction of ACADs can be divided into a reductive and an oxidative half-reaction. The reductive half-reaction is initiated by abstraction of the *pro*-*R*-α-proton of the acyl-CoA thioester by a conserved active site glutamate [4]. The concomitant hydride transfer of the *pro*-*R*-β-hydrogen to the N5 atom of the isoalloxazine ring of the FAD cofactor proceeds via an enolate-like intermediate, which forms a charge-transfer complex (CTC) with the FAD [5,6]. Although the substrate is rapidly converted into the CTC, no product is formed in the absence of ETF or another suitable electron acceptor [5]. Therefore, a round of catalysis can only be completed with the electron transfer from the CTC to ETF during the oxidative half-reaction. The oxidative half-reaction consists of two successive inter-protein one-electron transfers between reduced ACAD and two oxidized ETFs. This results in the re-oxidation of the ACAD bound FAD and yields two ETFs in the semiquinone state (ETFsq) [7].

In addition to reacting with ETF, ACADs are also able to use dioxygen as electron acceptor, albeit only as a side reaction and at a low rate [8]. There is a physiological need to keep the dioxygen reactivity of ACADs low. The oxidase side-reaction generates reactive oxygen species (ROS), such as hydrogen peroxide [9]. Furthermore, a transfer of electrons onto dioxygen instead of ETF would result in a loss of reducing equivalents that otherwise could fuel the electron transport chain, which would lower the ATP synthesis of cells.

Acyl-CoA oxidases (ACXs) are another family of flavoproteins that also oxidize α,β-carbon bonds in acyl-CoA thioesters. ACXs are phylogenetically, structurally, and mechanistically closely related to ACADs. However, in contrast to ACADs, ACXs do not require an ETF partner and directly use dioxygen as a final electron acceptor [4,10]. From an evolutionary, as well as a biochemical, point of view the differences in dioxygen reactivity between ACADs and ACXs are highly interesting. Yet, a clear understanding of the evolutionary and molecular determinants that cause the catalytic differences between both enzyme families is still lacking [9,11].

We recently converted (2*S*)-methylsuccinyl-CoA dehydrogenase (Mcd), a member of the ACAD enzyme family into a (2*S*)-methylsuccinyl-CoA oxidase (Mco) through three active site mutations [12,13]. The engineered Mco represents an interesting model system to investigate the factors affecting and steering dioxygen reactivity in flavoenzymes. In this study, we provide detailed insights into the mechanistic outcome of our engineering efforts and report on additional approaches to further engineer Mco from a dehydrogenase into a more efficient oxidase.

## 2. Results

### 2.1. The Oxidase Activity of Engineered Mco Cannot Be Improved by Increasing FAD’s Solvent Accessibility

We recently designed and realized a synthetic pathway for the conversion of carbon dioxide in vitro. One reaction in this synthetic pathway required an enzyme that could oxidize (2*S*)-methylsuccinyl-CoA into mesaconyl-CoA with dioxygen as electron acceptor [12]. Since no (2*S*)-methylsuccinyl-CoA oxidase was known, we sought to convert Mcd, a known member of the ACAD family, into an oxidase. We used structural modeling and multiple sequence alignments of ACADs and ACXs to identify three residues at the active site of Mcd that should allow to increase the reactivity of Mcd with dioxygen upon mutation (W315F, T317G and E377N). Of three single variant enzymes only the Mcd variant T317G (further referred as T317G) showed significant oxidase activity (*v*_max_ = 27.7 ± 0.7 mU mg^−1^; apparent *K*_M_ = 30.0 ± 2.5 µM), albeit very low compared to the dehydrogenase activity of the wild-type Mcd. Combination of all three mutations resulted in a variant with considerable oxidase activity (*v*_max_ = 97 ± 6 mU mg^−1^; apparent *K*_M_ = 27 ± 5 µM). We termed this triple-variant (2*S*)-methylsuccinyl-CoA oxidase (Mco) [12].

**Figure 1:**
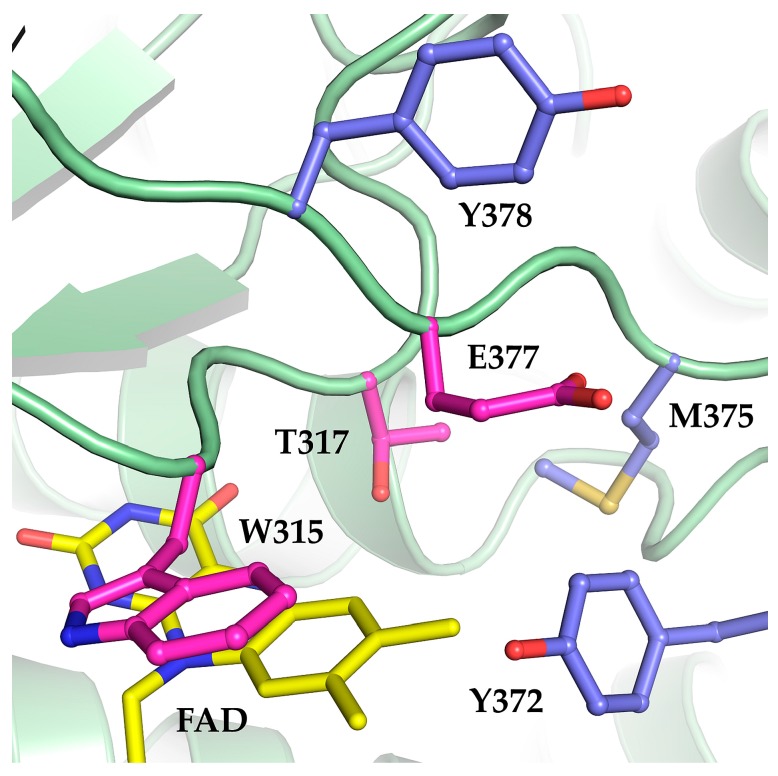
Structure model of Mcd from *Rhodobacter sphaeroides* based on the human short-chain acyl-CoA dehydrogenase (PDB ID: 2VIG). The engineered oxidase (Mco, green backbone) comprises three mutations: W315F, T317G and E377N (pink residues) around the FAD cofactor (yellow). We targeted three additional residues (Y372, M375, and Y378; blue residues) for site-directed mutagenesis with the aim to further increase oxidase activity by making the active site more solvent accessible.

Based on the success in developing Mco by rational design, we sought to extend this approach to find further Mco variants with improved oxidase activities. Using sequence alignments and structural modeling, we identified three additional residues (Y372, M375, and Y378) as targets. All three residues are located in the vicinity of the FAD cofactor. M375 and Y372 cover the isoalloxazine moiety of the FAD to shield it from solvent exposure (Figure 1). An increased solvation of the active site was proposed to increase reactivity towards dioxygen in ACADs due to stabilization of the formed superoxide [4,8,14]. Thus, we mutated Y372 and M375 to isoleucine and serine, respectively, because these smaller residues are partially conserved in other ACADs, according to a multiple sequence alignment (Figure S1). The same sequence alignment also revealed a conserved glycine in ACXs at the position of Y378 in Mcd. Although G378 does not appear to be in direct contact with the FAD according to structural models, the mutation Y378G was also suspected to increase dioxygen reactivity. The three mutations Y372I, M375S, and Y378G in the Mco variant background were assessed for oxidase activity. However, all three variants completely lost catalytic activity, suggesting that simply increasing accessibility for dioxygen is not a straight-forward approach to increase the oxidase reactivity in ACADs.

### 2.2. Mco Shows an Improved Oxidative Half-Reaction with Dioxygen

**Figure 2:**
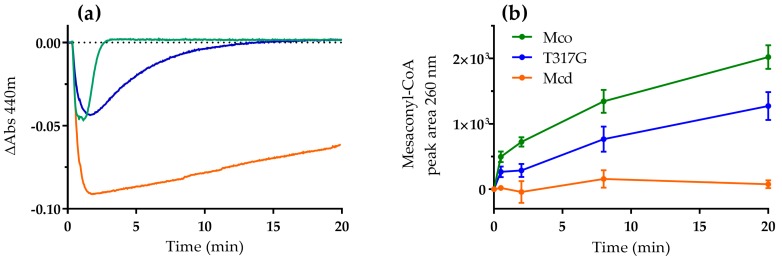
Reaction of Mcd (orange), T317G (blue) and Mco (green; 20 µM each) with dioxygen as electron acceptor. Reactions were started by the addition of ethylmalonyl-CoA (100 µM), which was converted in situ to (2*S*)-methylsuccinyl-CoA by ethylmalonyl-CoA mutase (0.5 µM). (**a**) The redox state of the enzyme-bound FAD was observed at 440 nm; and (**b**) product formation was analyzed by liquid chromatography (LC) with detection of mesaconyl-CoA at 260 nm. Error bars indicate the standard deviation of three assay replicates.

Next, we studied the catalytic behavior of Mcd and the oxidase variants T317G and Mco in the presence of dioxygen as only electron acceptor. We produced (2*S*)-methylsuccinyl-CoA in five-fold excess to the enzyme in situ [15] and followed the reaction of the FAD cofactor of the three enzymes at 440 nm. In order to observe both half-reaction in the presence of the high concentration of flavoprotein, the in situ substrate production rate was adjusted to be limiting. Overall, the three enzymes showed a similar biphasic reaction profile. Initially, there was a rapid decrease in absorbance indicating the reduction of the FAD cofactor. Subsequently, in a second phase, absorbance increased again, indicating the re-oxidation of the FAD to its pre-catalytic form (Figure 2a). While the first phase (reductive half-reaction) was comparable between all three variants, the re-oxidation rates of the FAD cofactor of the three enzymes differed widely.

In strong contrast to Mcd, which showed only little re-oxidation, the oxidase variants T317G and Mco were rapidly re-oxidized. In fact, T317G and Mco were fully oxidized after 17 min and 3 min, respectively. The re-oxidation rates were in line with the oxidase turnover number observed for T317G (27.7 mU mg^−1^) and Mco (97 mU mg^−1^) [12]. The re-oxidation of the two enzymes was accompanied by product (mesaconyl-CoA) formation, demonstrating that the catalytic cycle was indeed completed by both oxidase variants (Figure 2b). Compared to Mco and T317G, Mcd showed only very little product formation and its product formation rates did not correlate with the increase in absorbance over time. We observed a strong increase of free CoA in the Mcd assay over time, which was not observed for Mco and T317G (Figure S2). (*2S*)-Methylsuccinyl-CoA is an unusually unstable CoA ester [13] with a half-life of 24 min (Figure S3). Note that formation of the CTC is reversible and dependent on substrate concentrations. Thus, the very slow increase of Mcd absorbance in the assay over time is not due to completion of the catalytic cycle, but mainly due to the steadily decreasing (*2S*)-methylsuccinyl-CoA concentrations that in turn shift the equilibrium of the CTC slowly back to the oxidized FAD form.

To rule out that the observed oxidase activities of Mco (and T317G) were caused by the non-enzymatic reaction of reduced FAD with dioxygen in solution, we investigated whether reduced FAD can dissociate from the enzyme. The addition of oxidized FAD in two-fold excess over the substrate did not increase product formation by Mco under anaerobic conditions, indicating that reduced FAD cannot simply be exchanged by free oxidized FAD (Figure S4). Furthermore, when running a Mco reaction under oxic conditions on a 50 kDa filter, FAD could not be detected in the flow-through, providing additional evidence that FAD does not dissociate from the enzyme during catalysis (Figure S5). These results are in line with the proposed mechanism of ACAD and ACX according to which product can only be released after FAD is re-oxidized within the active site by a final electron acceptor.

Taken together, these experiments confirmed that the Mco and T317G directly react with dioxygen (with increasing reactivity from the single variant T317G to the triple variant Mco). Moreover, our results suggested that the transfer of the electrons from the FAD cofactor onto dioxygen is the rate-limiting step in the engineered oxidases.

**Figure 3:**
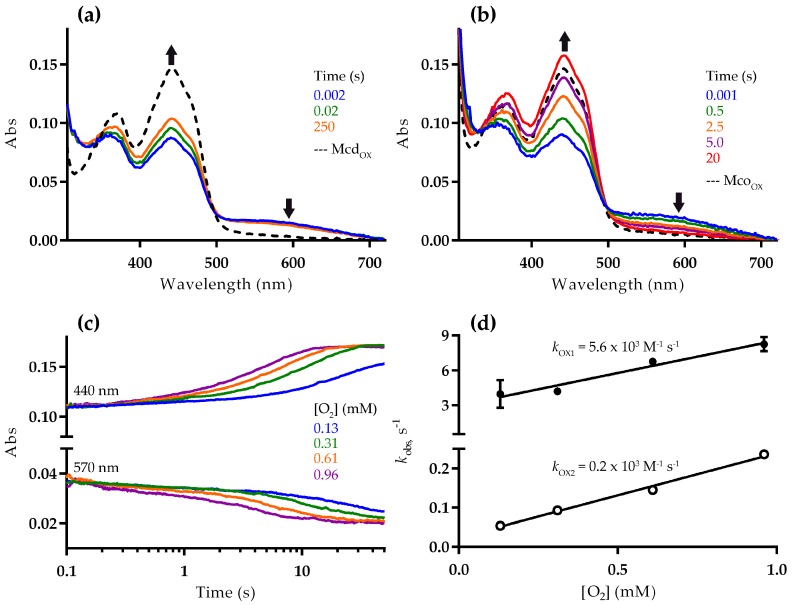
Reaction of reduced Mcd (**a**) and Mco (**b**–**d**) with dioxygen. Mcd or Mco was partially reduced by adding a stoichiometric amount of (*2S*)-methylsuccinyl-CoA under anaerobic conditions. Subsequently, the partially reduced enzyme was mixed with buffer containing various dioxygen concentrations using a stopped-flow spectrophotometer at 25 °C (0.13, 0.31, 0.61, and 0.96 mM dioxygen in the stopped-flow cell); (**a**,**b**) spectral changes showing Mcd (**a**) and Mco (**b**) re-oxidation with 0.96 mM dioxygen, as well as the spectrum of the fully-oxidized enzyme (broken line). Differences observed among the spectrum of the fully oxidized Mco and that of the re-oxidized Mco (20 s, red line) may due to the presence of product bound to re-oxidized enzyme (Figure S6) [18]; (**c**) stopped-flow traces recorded during Mco re-oxidation; and (**d**) stopped-flow traces at 440 nm were fit to a double exponential function to obtain the *k*_obs_ values versus dioxygen concentration.

To better understand the differences in the reductive and oxidative half reactions, we next studied the reaction of Mcd and Mco with dioxygen using a stopped-flow spectrophotometer. For these experiments, reduced enzyme was prepared in a vial under anaerobic conditions, by adding a stoichiometric amount of (2*S*)-methylsuccinyl-CoA. The reduction resulted in a decrease of absorbance at 440 nm and increase at 570 nm, consistent with formation of the CTC [16]. The first spectra recorded after stopped-flow mixing with dioxygen indicated that the enzymes were only partially reduced. This suggests that FAD reduction in Mcd and Mco is reversible, as described for other ACADs [16]. More specifically, the absorbance at 440 nm exhibited 65% and 57% of the fully oxidized Mcd and Mco, respectively (Figure 3a,b), which strongly points towards a slightly altered redox potential of the FAD cofactor in Mco through the three active site mutations. Alternatively, the mutations might favor binding of the product to the reduced Mco over binding of the substrate to the oxidized Mco, which could cause a perturbation of the equilibrium [17].

In the presence of 0.96 mM dioxygen, substrate-reduced Mcd was only re-oxidized to 76% (from 65% initially) after 250 s based on the observed spectra (Figure 3a). In contrast, Mco was fully re-oxidized after 20 s under the same conditions (Figure 3b). In both cases, no flavin semiquinone was observed. The re-oxidation rate decreased with lower dioxygen concentrations (Figure 3c). The corresponding stopped-flow traces at 440 nm were fit to a double exponential function, with an initial fast phase accounting for approximately 10% of the total absorption change. The observed rate constants of the two phases correlated linearly with the dioxygen concentration, giving a second-order rate constants of *k*_OX1_ = 5.6 × 10^3^ M^−1^ s^−1^ and *k*_OX2_ = 0.2 × 10^3^ M^−1^ s^−1^, respectively (Figure 3d). Although, the molecular basis for the biphasic re-oxidation of Mco by dioxygen is not clear, our results confirmed that the engineered oxidase function of Mco resulted from an improved oxidative half-reaction with dioxygen as an electron acceptor.

### 2.3. Mco Still Interacts with ETF, but at a Decreased Rate

**Figure 4:**
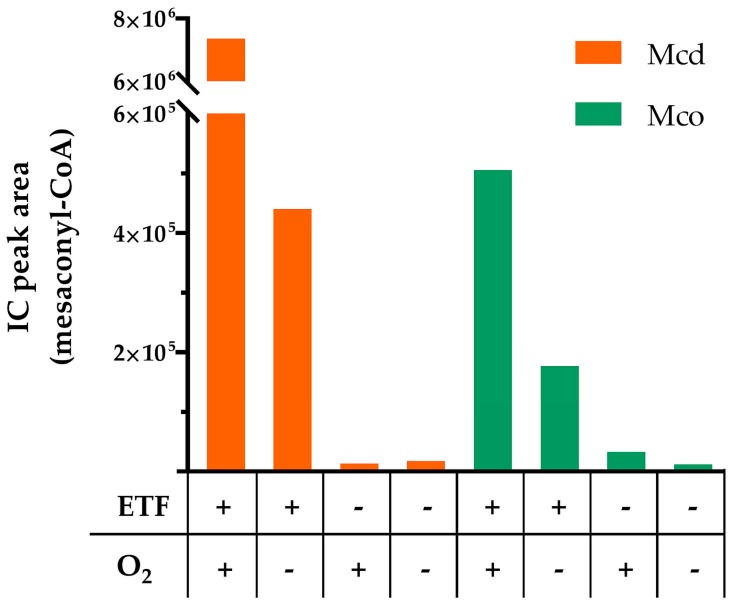
Representative analysis of product formation by Mcd and Mco after 1 h reaction time with ETF and/or O_2_. Assays were performed under aerobic (O_2_+) and anaerobic (O_2_−) conditions, in the presence of ETF (ETF+) or its absence (ETF−). Then, product formation was monitored by LC-MS. Reaction conditions: ETF 50 µM, air saturated solutions (235 µM O_2_ at 30 °C). 0.05 µM enzyme, 250 µM (2*S*)-methylsuccinyl-CoA.

We next assessed the ability of Mcd and Mco to interact with ETF as electron acceptor (Figure 4). Activity of Mcd was strictly dependent on the interaction with ETF. Under oxic conditions Mcd showed almost complete product formation with ETF, even though the concentration of the electron acceptor ETF was limiting in the assay. This can be explained by the spontaneous re-oxidation of ETFsq with dioxygen in solution over the running time of the experiment [19]. In contrast and as expected, product formation was limited under anoxic conditions.

Compared to Mcd, Mco did not require ETF for activity under oxic conditions. However, the enzyme produced more product with ETF in the presence of dioxygen and still showed activity with ETF under anoxic conditions. This suggests that Mco was still able to interact with ETF and retained some dehydrogenase activity. However, product formation was lower compared to Mcd, presumably because of a reduced rate of electron transfer to ETF. This is in line with the fact that the W315F mutation of Mco affected a tryptophan of the conserved isoalloxazine binding motif K-X-W/F-I-T [20], which is known to modulate electron transfer from ACADs to ETF. In human medium chain ACAD, mutation of the structurally conserved tryptophan to phenylalanine led to a 6-fold loss in catalytic efficiency of electron transfer [21]. Conclusively, the three mutations enabled the engineered Mco to accept dioxygen as an electron acceptor, but reduced the dehydrogenase activity with ETF as an electron acceptor.

**Figure 5:**
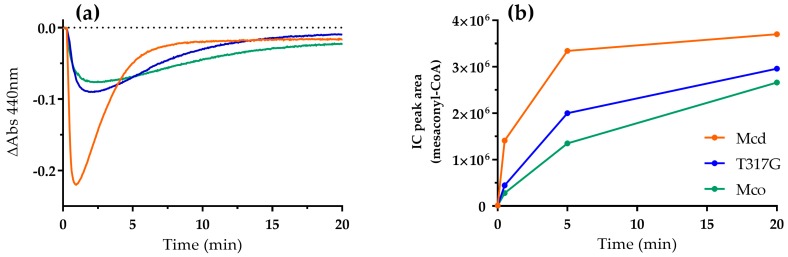
Reaction of Mcd (orange), T317G (blue) and Mco (green; 0.1 µM each) with ETF as electron acceptor (55 µM) under oxic conditions. Reactions were started by the addition of (2*S*)-methylsuccinyl-CoA (100 µM). (**a**) The redox state of the ETF-bound FAD was observed at 440 nm; and (**b**) samples were directly taken from the cuvette and analyzed for product (mesaconyl-CoA) by LC-MS.

Finally, we investigated the oxidative half-reaction of Mcd, Mco, and T317G with ETF under oxic conditions to understand electron transfer in the presence of the two (competing) electron acceptors in more detail. We provided ETF in 550-fold excess to each of the enzyme variants, which allowed for the direct observation of ETF-bound FAD. The reaction of the FAD cofactor showed two phases. First, a decrease in absorbance at 440 nm and a concomitant increase at 375 nm, indicating the generation of ETFsq (Figure 5a and Figure S7). Then a second, subsequent increase of absorbance followed, indicating re-oxidation of ETFsq by dioxygen. Mcd showed a fast electron transfer from the CTC to ETF that was accompanied by completion of product formation within 10 min (Figure 5b). On the other hand, T317G and Mco exhibited reduced electron transfer rates from the CTC to ETF. The initial slope of both enzymes was reduced by approximately 80% (Figure S8) and this apparent decrease in electron transfer to ETF was also reflected in lower product formation rates (Figure 5b). Taken together, these results suggested that even though T317G and Mco were still able to interact with ETF, their interaction was reduced compared to the WT, indicating a trade-off between oxidase and dehydrogenase activity in the engineered enzymes. In other words, while the reactivity with dioxygen increased with the mutations introduced into the Mcd scaffold, the efficiency of the canonical electron transfer to ETF became more defective.

## 3. Discussion

Despite extensive research, the molecular mechanisms governing the reactivity of flavoproteins towards dioxygen are still poorly understood [11]. ACXs and ACADs are particularly interesting in this regard, as they share high similarity in sequence, structure, and substrate specificity. However, they exhibit significantly different reactivity towards dioxygen. ACADs typically show a strongly reduced oxidase reactivity and transfer the electrons onto ETF. ACXs on the other hand use dioxygen as electron acceptor and produce hydrogen peroxide as a by-product. Here we studied Mcd, an ACAD homolog and engineered enzyme variants with increased oxidase activities to probe and understand the molecular factors that suppress and control dioxygen reactivity at the active site of ACADs.

Apparently one mutation at the active site (T317G) is enough to introduce significant oxidase reactivity into an ACAD scaffold, while three mutations yield an enzyme variant (Mco) that reacts at significant rate with dioxygen. The second-order rate constant for the reaction of Mco with dioxygen is 0.2 × 10^3^ M^−1^ s^−1^. However, oxidases and monooxygenases typically display second-order rate constants of 10^5^–10^6^ M^−1^ s^−1^, indicating that the electron transfer from FAD cofactor onto dioxygen is (still) rate limiting in the engineered enzymes [22]. Therefore, the dioxygen reactivity of Mco probably could be further improved, which might be achieved by either removing additional molecular factors suppressing oxidase reactivity in ACADs or introducing residues that increase oxidase activity directly.

De-solvation of the active site has been proposed to be crucial for suppressing oxidase activity in ACADs. This hypothesis was based on the observation that long-chain acyl-CoA oxidase and bacterial butyryl-CoA dehydrogenase, which feature more polar and solvent-accessible active sites react much more efficiently with dioxygen compared to medium-chain acyl-CoA dehydrogenase [10]. In agreement with this hypothesis, mutations W315F, T317G, and E377N that altogether increase solvent accessibility at the active site led to an increased oxidase activity in Mco. However, further mutagenesis of amino acids lining the FAD cofactor failed to increase oxidase activity, suggesting that solvent accessibility of the active site is an important but not sufficient criterion for reactivity towards dioxygen.

In this context one also has to consider that dioxygen has a rather hydrophobic character. Therefore, except for providing effective access to the reduced flavin cofactor, access channels should be lined with hydrophobic residues. Recently, the diffusion pathways for substrates and products of vanillyl alcohol oxidase (VAO) were analyzed computationally, which revealed dedicated dioxygen diffusion pathways [23]. Computational methods to analyze and predict trajectories by which dioxygen could reach the reduced flavin in flavoenzymes might be extremely valuable to guide future engineering efforts.

Another important principle in restricting the oxidase activity of dehydrogenases is gatekeeping. In alditol oxidase and related flavoenzymes, a dedicated residue was identified that strictly regulates dioxygen access to the active site of these enzymes [24,25]. While ACADs/ACXs are structurally distinct from VAO-type flavoenzymes, it is worth noting that T317 in Mcd is at a structurally similar position (lining the flavin cofactor close to the N5) and apparently plays a key role as a dioxygen gatekeeper.

In contrast to above mutations that mainly focus on removing factors that suppress dioxygen reactivity, other mutations might aim at actively promoting the oxidase functionality. An important example might be the newly introduced asparagine residue in the E377N variant. N377 could provide a hydrogen bond to stabilize formation of the superoxide anion intermediate, similar to the conserved residue N255 in peroxisomal short chain-specific oxidase from *Arabidopsis thaliana* (ACX4) [26]. Thus, increased dioxygen reactivity in Mco could also result from the loss of a negative charge by the E377N substitution. Additionally, in glucose oxidase and type I cholesterol oxidase dioxygen reactivity is increased by a factor of 1 × 10^4^ through protonation of a histidine close to N5 of FAD [27,28] and a single positively charged lysine in the active site of monomeric sarcosine oxidase is responsible for the activation of dioxygen [29]. Therefore, the introduction of a positively charged residue in the active site of Mco might further increase oxidase activity.

In summary, our results are able to explain the molecular basis for the successful implementation of an oxidase function in the scaffold of a dehydrogenase. However, they also revealed that introduction of mutations that increased oxidase reactivity apparently also caused a decreased dehydrogenase activity. Thus, our findings add to the general notion that increasing reaction rates for a specific substrate in enzymes is (very often) achieved at the expense of reaction promiscuity and provide at the same time guidelines for the rational engineering of ACADs and ACX in the future. In this respect it will be important to identify and probe additional factors in oxidases and dehydrogenases that determine reaction specificity. For example structural factors, such as N-terminal loops that exist in ACX4 and xanthine dehydrogenases/oxidases [26,30] and apparently prevent electron transfer to other electron acceptors besides dioxygen [31].

## 4. Materials and Methods

### 4.1. Cloning

Plasmids used in this study are described in Table 1. Oligonucleotides were synthesized by Eurofins Genomics (Ebersbach, Germany). The genes coding for ETF alpha subunit (*etfA*) and beta subunit (*etfB*) from *R. sphaeroides* were amplified using chromosomal DNA as template. For *etfA* two oligonucleotides introducing restriction sites (underlined) were designed upstream (5′-ATTAGGATCCGATGGCCGTTCTTCTGATT-3′; BamHI) and downstream (5′-CTACAAGCTTCGGTTCAGAGCTTGCCGGTCAG-3′; HindIII) of the gene. For *etfB* two oligonucleotides introducing restriction sites (underlined) were designed upstream (5′-TATACATATGAAGGTTCTGGTGCCTGTC-3′; NdeI) and downstream (5′-CATACTCGAGAACGGCCATCAGATCACC-3′; XhoI) of the gene. PCR was performed with Phusion^®^ High-Fidelity DNA Polymerase in GC buffer for 35 cycles, including denaturation for 60 s at 98 °C, annealing for 30 s at 55 °C and polymerization for 2 min at 72 °C. The PCR products were cloned into the pCDF-Duet1 vector for expression resulting in plasmid pTE392.

Point mutations were introduced by site-directed mutagenesis using QuikChange^®^ Site-Directed Mutagenesis Kit (Stratagene, San Diego, CA, USA) with slight adjustments. Plasmids and primers used for site-directed mutagenesis are listed in Table 2. The plasmid pTE22 (Mcd wild-type) and pTE813 (Mco), respectively, were used as templates. Double and triple mutants were generated by introducing each mutation in a successive QuikChange PCR (confirming correct mutagenesis after each step by sequencing). A 50 µL QuikChange reaction contained 60 ng of template plasmid, 0.25 µM forward and reverse primer, 200 µM dNTP, 5 µL 10× reaction buffer, 5% DMSO, and 1 µL Phusion polymerase (2 U/µL). Template plasmid was removed by DpnI digest (10 U) at 37 °C immediately after PCR amplification. Mutations were confirmed by sequencing.

### 4.2. Heterologous Protein Production and Purification

#### 4.2.1. Mcd Variants

Mcd and Mco variants were heterologously produced in *Escherichia coli* Rosetta (DE3) pLysS. 500 mL TB containing 10 µg/mL riboflavin and 100 µg/mL ampicillin (amp) was inoculated with freshly-transformed cells and incubated at 37 °C. After reaching an OD_600_ of 0.8 expression was induced by adding IPTG to a final concentration of 0.5 mM and the incubation temperature was lowered to 25 °C. Cells were harvested after 4 h by centrifugation (4500× *g*, 10 min) and resuspended in lysis buffer (500 mM NaCl, 20 mM Tris pH 8, 10% glycerol, protease inhibitor). If not used immediately, cell pellets were stored at −20 °C. The cell lysate obtained by sonication was clarified by centrifugation 50,000× *g* at 4 °C for 20 min. The supernatant was filtered through a 0.4 µm syringe tip filter (Sarstedt, Nümbrecht, Germany). Ni-affinity purification was performed with an Äkta FPLC system (GE Healthcare, Freiburg, Germany). The filtered soluble lysate was loaded onto a 1 mL Ni-Sepharose Fast Flow column (HisTrap FF, GE Healthcare) that had been equilibrated with 10 mL of buffer A (500 mM NaCl, 20 mM Tris pH 8). After washing with 20 mL 85% buffer A, 15% buffer B (500 mM NaCl, 20 mM Tris pH 8, 500 mM imidazole), the protein was eluted with 100% buffer B. The elution fractions containing purified protein were pooled and the buffer was exchanged to storage buffer (150 mM NaCl, 20 mM Tris pH 8) with a desalting column (HiTrap, GE Healthcare) Proteins were concentrated by ultrafiltration (Amicon Ultra 0.5 mL 30 kDa cut-off). Concentration of Mcd, which eluted with bound FAD cofactor was determined by the Bradford assay [32]. For all Mcd variants (Mco, T317G, Mco Y372I, Mco M375S, Mco M378G), which eluted with decreased FAD content, the concentration was determined on a NanoDrop 2000 Spectrophotometer (Thermo Scientific, Waltham, MA, USA) using the extinction coefficient at 280 nm, as calculated by Protparam [33]. The FAD cofactor was reconstituted by the addition of a two-fold excess of FAD over enzyme and incubation on ice for 30 min. Enzyme purity was confirmed by SDS-PAGE. The purified protein was stored in 50% glycerol at −20 °C.

#### 4.2.2. ETF

ETF was heterologously produced in *E. coli* BL21 (DE3) from the plasmid pTE392. Expression was performed as described for Mcd variants, however, cultures were harvested after overnight incubation. The Ni-affinity purification protocol was optimized, since the standard protocol did not yield detectable protein in the elution fractions. After loading, the column was washed first with 20 mL 100% buffer A, then 10 mL 2% buffer B, then with 5% buffer B and eluted with 100% buffer B. Even though only the α-subunit carries a His-tag, ETF eluted as a heterodimer (αβ). The elution fractions containing purified protein were pooled and the buffer was exchanged to storage buffer (150 mM NaCl, 20 mM Tris pH 8) with a desalting column (HiTrap, GE Healthcare). Proteins were concentrated by ultrafiltration (Amicon Ultra 0.5 mL 10 kDa cut-off). Concentration of ETF was determined by the Bradford assay [32]. Enzyme purity was confirmed by SDS-PAGE. The purified protein was stored in 50% glycerol at −20 °C.

### 4.3. Chemical Synthesis of Methylsuccinyl-CoA

101 mg (*S*)-methylsuccinic acid and 88 µL triethylamine were dissolved in 2 mL dry THF and put into an ice bath. 61 µL ethylchloroformate was slowly added to the reaction under excessive stirring. After 1 h, 100 mg CoA, dissolved in 5 mL 1 M H_2_CO_3_ (aq.) was added to the reaction. The reaction was monitored by mixing a small aliquot with Ellman’s reagent. After 1 h no free thiols were detected, indicating that the reaction had completed. The resulting solution was lyophilized and methylsuccinyl-CoA was purified by preparative liquid chromatography-mass spectrometry (LC-MS) with a methanol gradient in 25 mM ammonium formate buffer. The fractions containing the product were lyophilized and stored at −20 °C. For the use in enzyme assays, methylsuccinyl-CoA was dissolved in aq. HCl (pH 3) and the concentration was determined spectrophotometrically at 260 nm using the extinction coefficient of saturated CoA-thioesters (ε_260_ = 16.4 mM^−1^ cm^−1^). (2*S*)-Methylsuccinyl-CoA was assumed to be present at 50% mixture of the constitutional isomers of (2*S*)-methylsuccinyl-CoA and (3*S*)-methylsuccinyl-CoA. Therefore, the extinction coefficient was adjusted to 8.2 mM^−1^ cm^−1^.

### 4.4. Enzyme Assays

#### 4.4.1. LC and LC-MS-Based Assays

Reactions were run in 1.5 mL Eppendorf cups at 30 °C. For the experiments reported in Figure 4, the reactions contained 100 mM Tris-HCl pH 7.5, 50 mM KCl, 250 µM (2*S*)-methylsuccinyl-CoA or 50 µM ETF and 0.05 µM enzyme. For the experiments reported in Figures S3 and S4 the reactions contained 100 mM Tris-HCl pH 7.5, 50 mM KCl, 250 µM (2*S*)-methylsuccinyl-CoA, 10 µM enzyme and excess of FAD (500 µM) where indicated. 10 µL samples of the reaction were quenched in 2 µL 50% formic acid and precipitated proteins were removed by centrifugation at 17,000× *g*, 4 °C. For ensuing LC-MS analysis, the samples were diluted three-fold in water. LC-MS analysis was performed as described previously [34]. For assays analyzed by liquid chromatography (LC) only, detection was carried out at 260 nm. Anaerobic assays were performed in an anaerobic glove box (Coy Labs, Grass Lake, MI, USA). All reagents and enzymes stocks were made anaerobic by equilibrating in the glove box for 1 h prior to the assay.

#### 4.4.2. Spectrophotometric Assays

Spectrophotometric assays were carried out on Cary-60 UV-VIS spectrometer (Agilent, Waldbronn, Germany) at 30 °C with 10 mm quartz cuvettes (Hellma, Müllheim, Germany). Scanning kinetics were recorded in the range of 600 to 340 nm and single wavelength kinetics were extracted from those. In the ETF assays, the reactions contained 50 mM Tris-HCl pH 7.8, 100 µM (2*S*)-methylsuccinyl-CoA, 55 µM ETF, 0.1 µM Mcd in a final volume of 200 µL. In the enzyme reduction assays, the reactions contained 50 mM Tris-HCl pH 7.8, 100 µM ethylmalonyl-CoA, 20 µM Mcd, and 0.5 µM ethylmalonyl-CoA mutase (Ecm). Samples of 20 µL were collected after 30 s, 5 min, and 20 min and prepared for LC-MS as described above.

#### 4.4.3. Spectrophotometric Assay to Determine FAD Released after Mco Reduction

A control experiment was carried out to confirm that Mco does not release FAD after reduction. Mco was mixed with a stoichiometric amount of (*2S*)-methylsuccinyl-CoA (12 µM) in an Amicon Ultra-0.5 mL centrifugal filter device (cutoff 50 kDa). Immediately after adding the substrate (5 µL in a final volume of 200 µL), the sample was centrifuged for 10 min at room temperature (14,000× *g*). Next, a spectrum of the flow-through was recorded. For comparison, the same experiment was carried out using FAD (12 µM) instead of Mco. The assays were carried out in air saturated 50 mM potassium phosphate buffer, at pH 7.5. Mco was prepared in this buffer using an Econo-Pac 10DG desalting column (Bio-Rad, Hercules, CA, USA). Enzyme and FAD concentrations were determined based on their extinction coefficient at 440 nm and 450 nm, respectively (15.0 and 11.3 mM^−1^ cm^−1^, respectively). All spectra were recorded using a V-660 spectrophotometer (Jasco, Oklahoma City, OK, USA).

#### 4.4.4. Spectrophotometric Stopped-Flow Assay

The re-oxidation of Mcd and Mco was studied using the single-mixing mode of a SX20 stopped-flow spectrophotometer (Applied Photophysics, Surrey, UK). A xenon lamp and a photodiode array (PDA) detector were used. Assays were run in duplicate or triplicate by mixing equal volumes of reactants. All experiments were carried out in 50 mM potassium phosphate buffer at pH 7.5 and 25 °C. Solutions and the flow-circuit of the stopped-flow spectrophotometer were made anaerobic as previously described [35,36]. Spectral changes were recorded after mixing anaerobically-reduced enzyme with buffer containing various dioxygen concentrations (0.13, 0.31, 0.61, and 0.96 mM dioxygen in the stopped-flow cell). Reduced enzyme was prepared by mixing the enzyme with a stoichiometric amount of (*2S*)-methylsuccinyl-CoA in a vial under anaerobic conditions. For these experiments, the concentration of Mcd and Mco was determined based on the holoenzyme extinction coefficient at 440 nm, which is 14.8 and 15.0 mM^−1^ cm^−1^ in 20 mM Tris-HCl, 200 mM NaCl, 10% (*v*/*v*) glycerol at pH 7.7 and 25 °C, respectively. These values were calculated using SDS (0.5%), to release the FAD from the apoprotein, and a V-660 spectrophotometer (Jasco) as previously described [35]. All data were analyzed using the software Pro-Data (Applied Photophysics, Surrey, UK) or GraphPad Prism 6.05 (La Jolla, CA, USA).

## Figures and Tables

**Table 1:** Plasmids used in this study.

Plasmid	Backbone	Relevant Features	Source
pTE22	pET16b	*mcd* from *R. sphaeroides*, N-terminal His_10_-tag, T7 promoter, amp resistance marker	[13]
pTE392	pCDFDuet-1	*etfA* and *etfB* from *R. sphaeroides*, *etfA N*-terminal His_6_-tag, T7 promoter, streptomycin resistance marker	This work
pTE801	pET16b	Mcd from *R. sphaeroides* (T317G), *N*-terminal His_10_-tag,T7 promoter, amp resistance marker	[12]
pTE813	pET16b	*mcd* from *R. sphaeroides* (W315F, T317G, E377N), “Mco”; *N*-terminal His_10_-tag,T7 promoter, amp resistance marker	[12]

**Table 2:** Plasmids and primers used for site-directed mutagenesis.

Template	Mutation	Fw Primer 5′ to 3′	Rv Primer 5′ to 3′	Plasmid
pTE813	Y372I	CGAGATCGAGGTGCTGGGCAT CCGCGGCATGAAGAACTATG	CATAGTTCTTCATGCCGCGGA TGCCCAGCACCTCGATCTCG	pTE1218
pTE813	M375S	CTGGGCTACCGCGGCTCGAAG AACTATGAGATC	GATCTCATAGTTCTTCGAGCC GCGGTAGCCCAG	pTE1219
pTE813	M378G	GCATGAAGAACGGCGAGATCG GCTTC	GAAGCCGATCTCGCCGTTCTT CATGC	pTE1220

## Supplementary Materials

Supplementary Materials are available online.
