# Supplementary material for: Molecular Basis for Converting (2S)-Methylsuccinyl-CoA Dehydrogenase into an Oxidase
Source: Molecules. 2017 Dec 28;23(1):68. doi: 10.3390/molecules23010068 (PMC6017585; doi:10.3390/molecules23010068)
Supplement: Supplementary file 1 [file molecules-23-00068-s001.pdf]

Supplementary Materials:

Molecular basis for converting (2*S*)-methylsuccinyl-CoA dehydrogenase into an oxidase

Simon Burgener, Thomas Schwander, Elvira Romero, Marco W. Fraaije and Tobias J. Erb

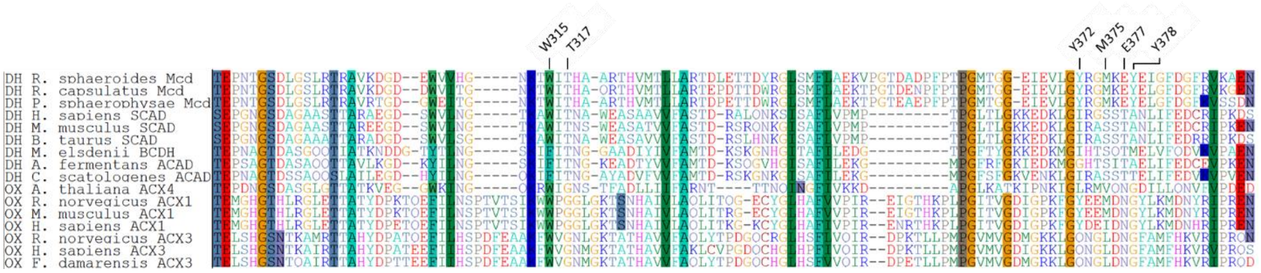

Figure S1. Multiple sequence alignment of ACAD and ACX. Indicated above are residues that were targeted for mutagenesis of Mcd from *R. sphaeroides*.

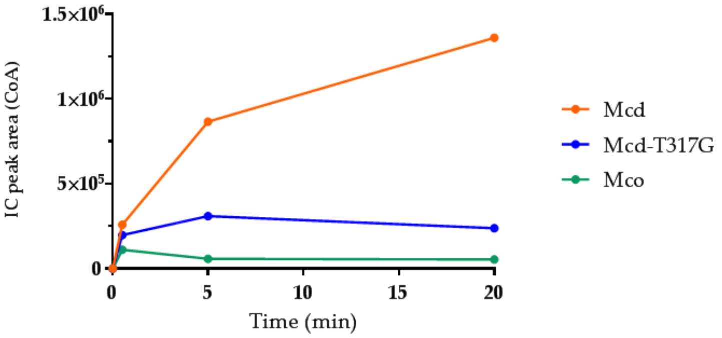

Figure S2. LC-MS analysis of free CoA in the reactions containing Mcd, T317G or Mco, in the presence of only dioxygen as electron acceptor.

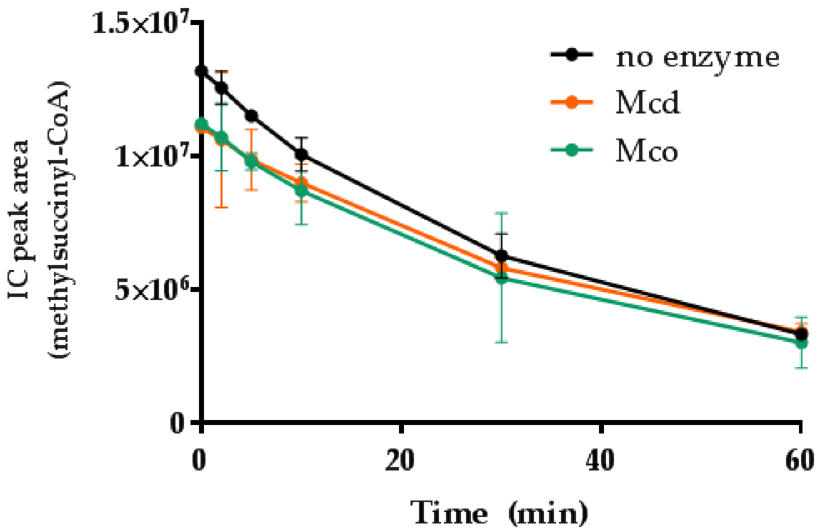

Figure S3. Spontaneous hydrolysis of (2*S*)-methylsuccinyl-CoA under anaerobic conditions in the presence of 10 μM enzyme (Tris-HCl 50 mM, pH 7.5, 30 °C). Error bars indicate 95% confidence interval from two independent replicates. The data was fit to a one phase decay equation and the half-life was determined to be 24 minutes.

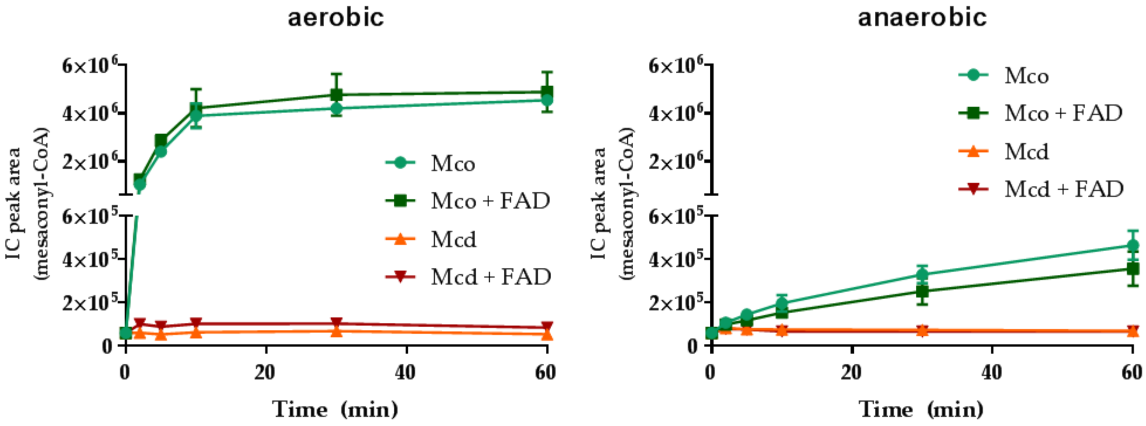

Figure S4. Product formation of Mcd and Mco (10 μM) with excess of FAD (500 μM) and (2*S*) methylsuccinyl-CoA (250 μM) under aerobic and anaerobic conditions. Error bars indicate standard deviation of two replicates of the assay. The low activity of Mco under anaerobic conditions is most likely due to residual dioxygen present in the assay.

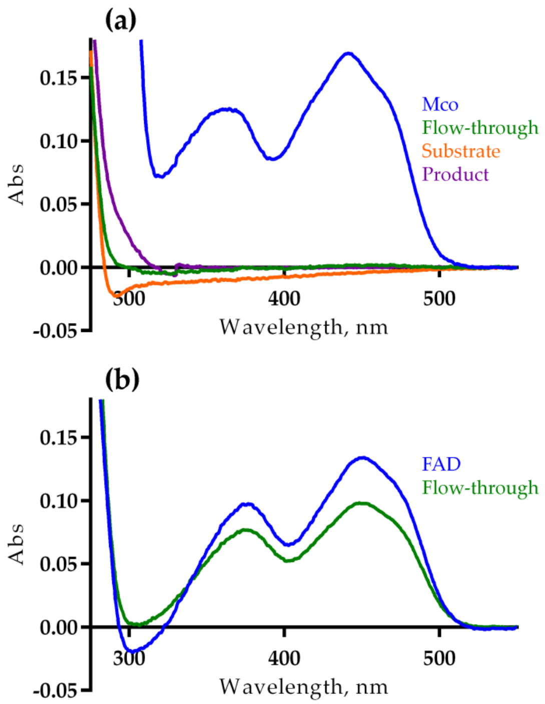

Figure S5. Determination of the amount of FAD released by reduced Mco. (a) A spectrum of Mco was recorded (blue line) before mixing Mco with a stoichiometric amount of methylsuccinyl-CoA in an Amicon Ultra-0.5 mL centrifugal filter device. Immediately after adding the substrate, the sample was centrifuged for 10 min at room temperature. Next, a spectrum of the flow-through was recorded (green line). The spectrum of methylsuccinyl-CoA and mesaconyl-CoA (both 12 μM) in the absence of enzyme are also shown (orange and purple lines, respectively). The high absorbance observed for the flow-through at wavelengths higher than 300 nm is presumably due to the presence of either unreacted substrate or product. (b) For comparison, the experiment described in (a) was carried out using FAD instead of Mco. The spectrum of FAD before adding the substrate (blue line) and that of the flow-through (green line) are shown.

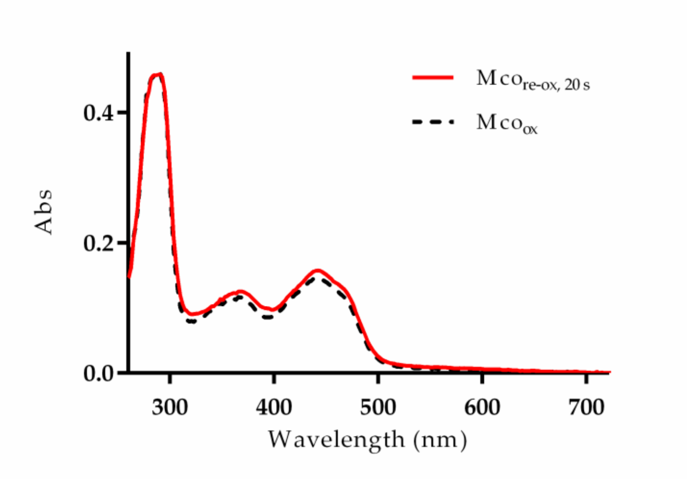

Figure S6. Comparison of the spectrum observed for the fully oxidized Mco and the re-oxidized Mco. The spectrum of the fully oxidized Mco (Mco$_{ox}$, broken line) was recorded in the absence of either substrate or product. Next, fully oxidized Mco was partially reduced by adding a stoichiometric amount of methylsuccinyl-CoA under anaerobic conditions. The resulting reduced enzyme was mixed with buffer containing dioxygen using a stopped-flow spectrophotometer at 25 °C (0.96 mM dioxygen in the stopped-flow cell). The spectrum recorded after 20 s of enzyme re-oxidation is shown (Mco$_{re\text{-}ox}$, 20 s, red line). The absorbance at 280 nm for both spectra is the same, confirming that the samples present the same enzyme concentration. However, the spectrum of the re-oxidized Mco exhibits higher absorbance at 440 nm than that for the fully oxidized enzyme among other differences. This fact may due to the presence of product bound to re-oxidized enzyme [1].

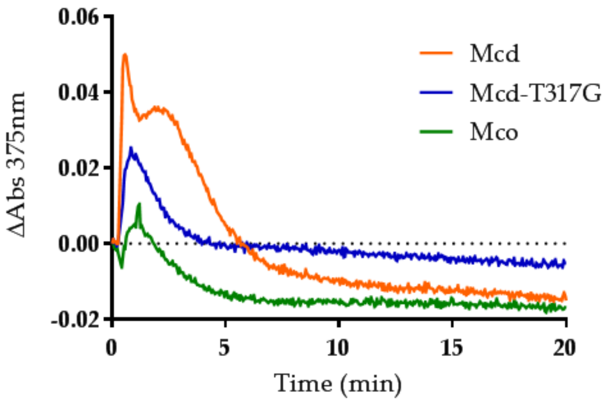

Figure S7. Reaction of Mcd, T317G and Mco (0.1 µM each) with ETF as electron acceptor (55 µM) under aerobic conditions. Reactions were started by the addition of (2*S*)-methylsuccinyl-CoA (100 µM). The redox state of the ETF-bound FAD was observed at 375 nm.

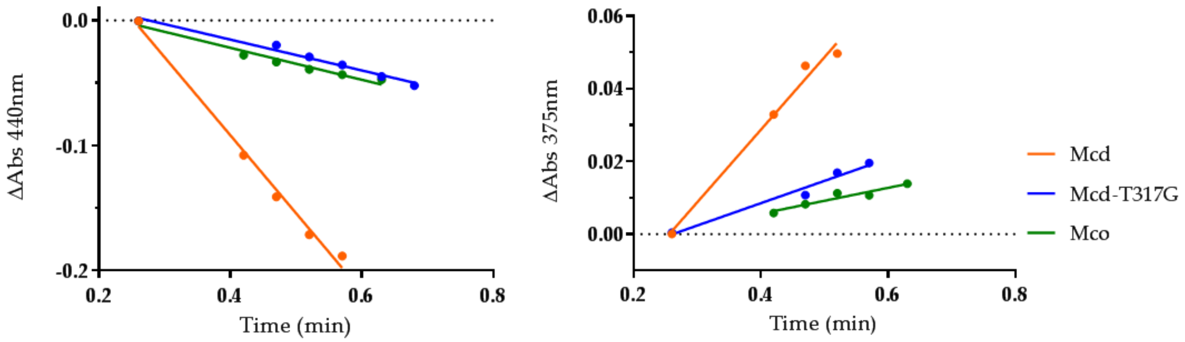

enzyme	440 nm Δabs/min	375 nm Δabs/min
Mcd	-0.62 ± 0.03	0.20 ± 0.02
Mcd-T317G	-0.12 ± 0.01	0.06 ± 0.01
Mco	-0.13 ± 0.01	0.04 ± 0.01

Figure S8. Linear regression of the initial slopes in the ETF reduction assay in Figure 5 and Figure S6.

References

1. Engel, P. C.; Massey, V., Green butyryl-coenzyme A dehydrogenase. An enzyme-acyl-coenzyme A complex. *Biochem. J.* **1971,** 125, 889-902.
